# Evaluation of a Method for Standardized Antimicrobial Susceptibility Testing with *Mycoplasma hyorhinis* Field Isolates

**DOI:** 10.3390/microorganisms11122881

**Published:** 2023-11-29

**Authors:** Lisa Käbisch, Anne-Kathrin Schink, Doris Hoeltig, Jutta Verspohl, Miklós Gyuranecz, Joachim Spergser, Corinna Kehrenberg, Stefan Schwarz

**Affiliations:** 1 Institute of Microbiology and Epizootics, Centre for Infection Medicine, School of Veterinary Medicine, Freie Universität Berlin, 14163 Berlin, Germany; lisa.kaebisch@fu-berlin.de; 2 Veterinary Centre for Resistance Research (TZR), School of Veterinary Medicine, Freie Universität Berlin, 14163 Berlin, Germany; 3 Institute for Veterinary Food Science, Department of Veterinary Medicine, Justus Liebig University Giessen, 35392 Giessen, Germany; corinna.kehrenberg@vetmed.uni-giessen.de; 4 LDG Laboratory Diagnostics Germany GmbH, 27472 Cuxhaven, Germany; a.schink@labdiag-de.com; 5 Division for Pigs, Farm Animal Clinic, School of Veterinary Medicine, Freie Universität Berlin, 14163 Berlin, Germany; doris.hoeltig@fu-berlin.de; 6 Institute of Microbiology, University of Veterinary Medicine Hannover, Foundation, 30173 Hannover, Germany; jutta.verspohl@tiho-hannover.de; 7 HUN-REN Veterinary Medical Research Institute, H-1143 Budapest, Hungary; gyuranecz.miklos@vmri.hu; 8 MolliScience Kft., H-2051 Biatorbágy, Hungary; 9 Institute of Microbiology, University of Veterinary Medicine, 1210 Vienna, Austria

**Keywords:** antimicrobial susceptibility testing (AST), broth microdilution, standardized methodology, *Mycoplasma hyorhinis*

## Abstract

Organizations like the Clinical and Laboratory Standards Institute (CLSI) or the European Committee of Antimicrobial Susceptibility Testing (EUCAST) provide standardized methodologies for antimicrobial susceptibility testing of a wide range of nonfastidious and fastidious bacteria, but so far not for *Mycoplasma* spp. of animal origin. Recently, a proposed method for the standardized broth microdilution testing of *Mycoplasma hyorhinis* using commercial Sensititre microtiter plates was presented. In this study, we evaluated this broth microdilution method with 37 field isolates and tested their susceptibility toward the following antimicrobial agents: doxycycline, enrofloxacin, erythromycin, florfenicol, gentamicin, marbofloxacin, tetracycline, tiamulin, tilmicosin, tulathromycin, and tylosin. The isolates originated from different countries, isolation sites, and years. The broth microdilution method was carried out using a modified Friis broth as the culture and test medium. For macrolides and lincosamides, a bimodal distribution with elevated MIC values could be observed for almost half of the tested field isolates, deducing reduced susceptibility toward these substances. With a recently published protocol, we were able to test a variety of field isolates, and consistent data could be obtained. Using this method, monitoring studies of *Mycoplasma hyorhinis* isolates can be carried out in a comparable manner, and the observed susceptibility profiles can be screened for possible changes in MIC values in the future.

## 1. Introduction

*Mycoplasma* (*M*.) *hyorhinis* is a porcine pathogen of ubiquitous origin [1,2]. As a facultative pathogen, the clinical picture of the infected porcine host may vary from severe systemic infections to polyserositis and arthritis in nursery piglets or chronic arthritis, conjunctivitis, and meningitis in older pigs [1,2,3,4,5,6,7]. Nevertheless, *M. hyorhinis* is often also isolated from pigs showing no clinical symptoms. Because of the great variety of clinical manifestations, the economic impact of an infection with *M. hyorhinis* is difficult to enumerate. To the best of our knowledge, there is no literature available estimating the financial losses specifically for a *M. hyorhinis*-infected herd. Furthermore, while animals with clinical signs will be individually treated, in severe cases, a farm-specific vaccination is implemented; animals that need to be euthanized, especially within the finishing phase, will represent the greatest losses for farmers [8].

Due to the lack of a cell wall, *Mycoplasma* spp. are intrinsically resistant to β-lactams and other cell-wall-targeting antimicrobial agents. The lack of specific enzymes within the folic acid metabolism pathway also renders antimicrobial agents targeting this specific pathway, such as sulfonamides and trimethoprim, ineffective [9,10,11,12,13]. Nonetheless, several classes of antimicrobial agents are available for the treatment of *M. hyorhinis* infections, and various studies have shown differing antimicrobial susceptibility profiles of *M. hyorhinis* field isolates in the past [10,13,14,15,16,17,18,19,20]. However, since different antimicrobial susceptibility testing (AST) protocols and methodologies have been used in previously published studies, the respective AST data are difficult to compare [21,22].

For all *Mycoplasma* spp. of animal origin, the most effective antimicrobial classes are fluoroquinolones, macrolides, pleuromutilins, and tetracyclines [13,14,23]. Reduced efficacies toward several antimicrobial agents have been observed during the last decades, involving both human as well as veterinary *Mycoplasma* spp. [5,10,14,15,16,17,20,24,25,26]. In *Mycoplasma* spp., resistance development is mainly conferred by chromosomal mutations, such as the well-described point mutation within the 23S rRNA, conferring resistance to 14-membered macrolides [15,18,19,23,25,27,28,29,30,31].

Because of the observations of reduced efficacy toward certain antimicrobial agents, in addition to increased monitoring of antimicrobial resistance among bacterial species of animal origin and efforts toward reducing the usage of antimicrobial agents in farm animals, as implemented by many governments, a standardized AST methodology for monitoring studies needs to be established [21]. For a standardized AST methodology, organizations, including the Clinical and Laboratory Standards Institute (CLSI) or the European Committee on Antimicrobial Susceptibility Testing (EUCAST), as well as more local authorities, such as the American Food and Drug Administration (FDA), the German Deutsches Institut für Normung (DIN), or the French Société Française de Microbiologie (SFM), provide AST standards or guidelines for a wide variety of nonfastidious and fastidious bacteria of human or animal origin. However, until now, there has been no consensus method for *Mycoplasma* spp. of animal origin.

The use of diverging AST methodologies generally leads to inconsistent data, rendering the obtained results incomparable and, when considering monitoring studies, unusable. For *M. hyorhinis*, the available literature indicates several methodologies, whereas most studies agree on using the broth microdilution method [10,14,15,16,20,25]. On the other hand, a great variety of broth media is used for AST, wherefore the minimal inhibitory concentrations (MICs) of the tested isolates differ and are consequently incomparable or lead to misinformation, especially concerning the in vivo activity of antimicrobial agents against *M. hyorhinis* infections [9,14,15,16,18,19,32,33,34]. In addition, monitoring of the emergence of a decreased susceptibility profile of *M. hyorhinis* is not possible, not only due to the diverging methodology but also because of the lack of established interpretive criteria [21,22]. Until now, no species-specific clinical breakpoints have been available that allow the classification of *M. hyorhinis* isolates as susceptible, intermediate, or resistant to the tested antimicrobial agents.

We have recently published a broth microdilution method suitable for standardization including quality control (QC) strains for AST of *M. hyorhinis* [35,36]. In this study, we further evaluated this methodology by applying it to a variety of *M. hyorhinis* field isolates.

## 2. Materials and Methods

### 2.1. Bacterial Strains

The type strains *M. hyorhinis* DSM 25591 (ATCC 17981), *Enterococcus faecalis* DSM 2570 (ATCC 29212), and *Staphylococcus aureus* DSM 2569 (ATCC 29213) were all purchased from the German Collection of Microorganisms and Cell Cultures GmbH (DSMZ, Braunschweig, Germany). A total of 37 *M. hyorhinis* field isolates originating from Austria, Hungary, and Germany were included in the study. They were all obtained from diagnostic samples taken from different body sites (joint or synovial fluid, serosa, lung, nasal cavity) submitted for microbiological examination between 2002 and 2021 (Table 1). After arrival at our laboratory, they were cultured in modified Friis broth [35], and their identity was confirmed by a *Mycoplasma*-specific nested PCR and subsequent sequencing of the PCR products [37].

### 2.2. Media

The isolates were cultured and tested in modified Friis broth, as previously described [35]. The modifications, with regard to the composition of the medium, as provided by the DSMZ, included (i) an increase in porcine serum to ensure the survival of the bacteria during the freeze–thaw cycle without the addition of cryopreservatives, (ii) the doubling of the phenol red solution to intensify the color of the broth and make color changes more visible, and (iii) the adaptation of the amount of yeast extract solution and deionized water to maintain the equilibrium of the provided nutrients [38].

Approximately 176 mL of the modified Friis broth, composed of 0.82 g of porcine Brain and Heart Infusion (BHI) (Sigma-Aldrich Chemie GmbH, Taufkirchen, Germany), 0.87 g of Difco Mycoplasma PPLO Broth w/o CV (Becton Dickinson (BD), Franklin Lakes, NJ, USA), 50 mL of filter sterilized Hank’s Balanced Salt Solution (as provided by [39]), and 78 mL of deionized water, was prepared. Before autoclaving at 121 °C, 1 bar for 15 min, the pH was adjusted to 7.4. After cooling, 40.6 mL of heat-inactivated porcine serum (Biowest SAS, Nuaillé, France), 4.49 mL of 25% autoclaved yeast extract solution (Carl Roth GmbH + Co. KG, Karlsruhe, Germany), 0.361 mL of a 1% filter sterilized phenol red solution (phenol red sodium salt, Carl Roth GmbH), and 0.285 mL of autoclaved deionized water were added. For solid media, 1% agar-agar (Carl Roth GmbH) was added before autoclaving.

### 2.3. Culture, Storage, and Quantification of M. hyorhinis

For each isolate, a minimum of two subcultures was produced. An aliquot of 500 μL of the primary culture was diluted into 5 mL of freshly prepared modified Friis broth. The cultures were incubated at 37 °C, 7.5% CO_2_, until a visible color change occurred, or for up to 14 days. The second subculture was quantified (see description below), aliquoted, and stored at −80 °C until further use.

The quantification of the broth culture was carried out according to CLSI recommendations and as previously described [35,40]. A 10-fold serial dilution over five steps was set up, and finally, 20 μL of each dilution step, as well as the original broth culture, were dropped onto modified Friis agar, air-dried, and then incubated at 37 °C, 5% CO_2_, for three to 14 days, until individual colonies could be detected. Agar plates that did not show colonies after a maximum of 14 days of incubation were discarded as negative, and cultivation had to be repeated. After incubation, 30 to 300 individual colonies were counted, and the number of CFU/mL of the initial broth culture was calculated.

For controlling the correct inoculum size for each AST approach, this method of quantification was applied. Since the expected inoculum density was less than the respective broth culture, only two dilution steps were carried out.

### 2.4. Antimicrobial Susceptibility Testing of M. hyorhinis

For quality control purposes of each experiment, fresh overnight cultures of the quality control strains (QCs) *E. faecalis* DSM 2570 and *S. aureus* DSM 2569 were used as previously described [36]. The inoculum suspensions were prepared according to CLSI standards [41]. We prolonged the incubation periods to account for the slower growth rate of *M. hyorhinis*, and the modified Friis broth was used as the test medium instead of the cation-adjusted Mueller Hinton broth [36]. In addition, the type strain *M. hyorhinis* DSM 25591 was used to control for *Mycoplasma* growth [35].

The inoculum of all *M. hyorhinis* strains (type strain and field isolates) was calculated to be 1 × 10^5^ CFU/mL, with an acceptable range between 5 × 10^4^ CFU/mL and 5 × 10^5^ CFU/mL, as described in the CLSI document for AST of human mycoplasmas [40]. The calculated volume of the slowly thawed frozen stock culture was transferred into the prewarmed (room temperature) modified Friis broth.

The prepared inoculum suspensions were preincubated for 2 h at 37 °C, 7.5% CO_2_, to restore fitness of the freshly thawed bacteria. This step of preincubation is equivalent to the fresh overnight culture of nonfastidious bacteria to generate a metabolically active culture.

From the preincubated inocula, 50 μL of the inoculum suspension was transferred into each well of the commercially available Sensititre microtiter plates (Thermo Fisher Scientific, Waltham, MA, USA) as described in the national resistance monitoring program GE*RM*-Vet [42]. The microtiter plates were sealed with an adhesive foil and incubated at 37 °C, ambient air, until a color change in the growth controls, indicative of bacterial growth, was observed. If no color change occurred after a maximum incubation time of 14 days, the microtiter plates were discarded, and the AST was denoted as invalid and had to be repeated. The following antimicrobial agents were investigated: clindamycin (0.03–64 mg/L), doxycycline (0.06–128 mg/L), enrofloxacin (0.008–16 mg/L), erythromycin (0.015–32 mg/L), florfenicol (0.12–256 mg/L), gentamicin (0.12–256 mg/L), marbofloxacin (0.008–16 mg/L), tetracycline (0.12–256 mg/L), tiamulin (0.03–64 mg/L), tilmicosin (0.06–128 mg/L), tulathromycin (0.06–32 mg/L), and tylosin (0.06–128 mg/L).

The MIC values of the *M. hyorhinis* type strain and field isolates were recorded as described earlier [35]. Since growing *M. hyorhinis* does not cause turbidity in broth media, the indicator phenol red was used in order to evaluate the microtiter plates visually. Therefore, the MIC was defined as the first well, where the color change from red (no growth) to yellow (growth) was incomplete. Trailing (orange) was observed but defined as no growth. The microtiter plates were examined daily with the unaided eye by the same person. When the growth controls showed the expected color change and the MIC values were recorded, the microtiter plates were incubated for an additional 24 h, and the results were recorded again. The endpoint of the AST was determined as the point when the color change was complete, and no major changes (more than ± one dilution step compared to the previous recording) of the recorded MIC values were observed. When major changes were noted, and consistency in the repeated readouts could not be achieved, the test was termed invalid and was repeated.

## 3. Results and Discussion

The antimicrobial agents were chosen according to therapeutic interest and the recommendations given by the CLSI, considering routine testing and relevance for swine [43]. Porcine mycoplasmas are expected to be susceptible to aminoglycosides, fluoroquinolones, phenicols, pleuromutilins, and tetracyclines [1,44]. Macrolide susceptibility is known to be variable [13]. Clindamycin was chosen as a representative for the class of lincosamides [43].

The QC strains *E. faecalis* DSM 2570 and *S. aureus* DSM 2569, as well as the type strain *M. hyorhinis* DSM 25591, were applied as published elsewhere, and MIC results were compliant with the published data [35,36].

The acquired MIC values of the 37 field isolates are shown in Table 2. Individual incubation times ranged from 72 h to 10 days.

For the antimicrobial agents gentamicin (0.25–2 mg/L), enrofloxacin (0.25–2 mg/L), marbofloxacin (0.25–2 mg/L), florfenicol (≤0.12–4 mg/L), erythromycin (2—≥64 mg/L), tiamulin (≤0.03–0.5 mg/L), doxycycline (≤0.06–1 mg/L) and tetracycline (≤0.12–2 mg/L), unimodal (i.e., with one peak) distributions of the MIC values were observed. A bimodal distribution (i.e., with two peaks) was noticed for the antimicrobial agents clindamycin (0.06–0.5 mg/L and 8–64 mg/L) and tilmicosin (0.12–2 mg/L and ≥256 mg/L). Tulathromycin and tylosin showed multimodal distributions (i.e., with multiple peaks).

In unimodal MIC distributions, as seen for tiamulin, doxycycline, and tetracycline, the respective bacteria represent the wild-type subpopulation. The isolates within the wild-type subpopulation are defined as those with no phenotypically detectable mechanisms of acquired resistance or reduced susceptibility for the antimicrobial agent being evaluated [45]. As a consequence, these wild-type isolates commonly display rather low MIC values. In our study, we also detected unimodal MIC distributions of *M. hyorhinis* isolates that displayed higher MICs of gentamicin, enrofloxacin, and marbofloxacin. As long as no clinical breakpoints are available that are applicable to *M. hyorhinis* and the aforementioned antimicrobial agents, as well as no information is provided about resistance-mediating mutations or resistance genes in the respective isolates, it is not possible to say whether the “wild-type” definition applies to these isolates. In bimodal and multimodal MIC distributions, usually, the subpopulation with the highest MIC values represents the non-wild-type subpopulation. It comprises isolates with presumed or known mechanisms of acquired resistance or reduced susceptibility for the antimicrobial agent being evaluated [45]. In our study, the same 15 *M. hyorhinis* isolates displayed tilmicosin MICs of ≥256 mg/L, tulathromycin MICs of 8–≥64 mg/L, tylosin MICs of 8–≥256 mg/L, erythromycin MICs of ≥64 mg/L, and clindamycin MICs of 8–64 mg/L.

According to the literature, *M. hyorhinis* is highly susceptible to gentamicin, fluoroquinolones, lincosamides, macrolides (except for erythromycin), pleuromutilins, and tetracyclines [13,16,46]. For most of these classes of antimicrobial agents, our results confirm these observations. However, high MIC values for macrolides and lincosamides were observed. About 40% (15/37) of the tested clinical isolates showed elevated MIC values (≥8 mg/L), occasionally above the highest available test concentrations. Reduced susceptibility to lincosamides and macrolides has also been observed in earlier studies [15,17,19]. Due to differing methodologies, the results are not directly comparable with those of other studies. However, the observed distributions of the tested field isolates in this study are mostly in accordance with other recent studies [14].

The high MICs observed for macrolides and lincosamides among the *M. hyorhinis* have serious clinical implications. Although clinical breakpoints that classify these isolates as “resistant” are currently not available, macrolides and lincosamides should not be used for therapeutic applications in these cases. The whole genome sequence of the type strain *M. hyorhinis* DSM 25591 showed the presence of a G2057A (*Escherichia coli* numbering) transition, which is known to confer resistance to 14-membered macrolides [27]. Földi and coworkers recently identified an A2058G transition via a mismatch amplification mutation assay in *M. hyorhinis* field isolates [47]. Moreover, an A2059G transition has been described in Japanese *M. hyorhinis* isolates [19].

When comparing the distribution of MIC values, the MIC_50_ and the MIC_90_ values with regard to time intervals (2002–2012 and 2013–2021), only minor trends in either direction could be observed (Table 3 and Table 4). Due to the small number of analyzed field isolates, statistical analysis was forgone. For the antimicrobial agents doxycycline, tetracycline, tiamulin, and erythromycin, no change could be observed. The distribution of MIC values of the tested field isolates between the two time intervals was comparable, and the MIC_50_ and MIC_90_ values were the same for both time periods. The MIC values of marbofloxacin were the only values that slightly increased from 2002–2012 to 2013–2021. This observation was also confirmed by an increase in both the MIC_50_ and MIC_90_ values. For the other antimicrobial agents (enrofloxacin, florfenicol, clindamycin, tilmicosin, tulathromycin, and tylosin), the recorded MIC values decreased, which was also observed in a decrease in the MIC_50_ values by one to five dilution steps for all aforementioned antimicrobial agents. The most pronounced decrease was observed for tylosin. A decrease in the MIC_50_ values indicates a larger number of isolates with lower MIC values. For tilmicosin, an at least two-fold decrease in the MIC_90_ values was also observed. The occurrence of bimodal and multimodal distributions, though not surprising, emphasizes the importance of the recognition that there are isolates with decreased susceptibility circulating within pig farms.

The observations in this study underline the importance of using a harmonized methodology and corresponding monitoring studies to register changes in antimicrobial susceptibility early on. In order to perform comparable surveillance studies, AST needs to be conducted in a harmonized manner. The recently proposed broth microdilution method appears to be a suitable AST method for *M. hyorhinis* field isolates of varying origins [35].

## 4. Conclusions

With increased accounting of antimicrobial substance application in farm animals, as well as ongoing monitoring of respective bacterial pathogens and their antimicrobial susceptibility profiles, the availability of a standardized methodology is of utmost importance. The recently published harmonized method was used to successfully test a variety of 37 *M. hyorhinis* field isolates. For most isolates, MIC values within the lower concentration ranges of the tested antimicrobial substances were observed. A subset of field isolates showed elevated MIC values for antimicrobial agents within the classes of macrolides and lincosamides. Although further studies are needed to establish clinical breakpoints, this is the first study confirming that the previously described broth microdilution AST method is not only suitable for the type strain *M. hyorhinis* DMS 25591 (ATCC 17981) but also for *M. hyorhinis* field isolates from various sources.

## Figures and Tables

**Table 1:** Background information of *M. hyorhinis* type strain and field isolates used in this study.

Isolate ID	Origin		
	Country	Year	Tissue
*M. hyorhinis* DSM 25591 type strain	Unknown	1955	Nasal cavity
*M. hyorhinis* 906L02	Austria	2002	Lung
*M. hyorhinis* 1089L03	Austria	2003	Lung
*M. hyorhinis* 2158N03	Austria	2003	Nasal cavity
*M. hyorhinis* 259L08	Austria	2008	Lung
*M. hyorhinis* 2618L08	Austria	2008	Lung
*M. hyorhinis* 82L09	Austria	2009	Lung
*M. hyorhinis* 265L09	Austria	2009	Lung
*M. hyorhinis* 1191L09	Austria	2009	Lung
*M. hyorhinis* 386S09	Austria	2009	Serosa
*M. hyorhinis* 1255L10	Austria	2010	Lung
*M. hyorhinis* 1533S10	Austria	2010	Serosa
*M. hyorhinis* 158L11	Austria	2011	Lung
*M. hyorhinis* 207L11	Austria	2011	Lung
*M. hyorhinis* 507S11	Austria	2011	Serosa
*M. hyorhinis* 67L12	Austria	2012	Lung
*M. hyorhinis* 12048421L13	Austria	2013	Lung
*M. hyorhinis* 3174S13	Austria	2013	Serosa
*M. hyorhinis* 3081L13	Austria	2013	Lung
*M. hyorhinis* 1606S14	Austria	2014	Serosa
*M. hyorhinis* 3631L14	Austria	2014	Lung
*M. hyorhinis* 3661N14	Austria	2014	Nasal cavity
*M. hyorhinis* 1191L15	Austria	2015	Lung
*M. hyorhinis* 1438L15	Austria	2015	Lung
*M. hyorhinis* 57L15	Austria	2015	Lung
*M. hyorhinis* 3565L16	Austria	2016	Lung
*M. hyorhinis* MycSu 75	Hungary	2017	Synovial Fluid
*M. hyorhinis* MycSu 111	Hungary	2017	Lung
*M. hyorhinis* 3044/1/19	Germany	2019	Joint
*M. hyorhinis* 4812/1/19	Germany	2019	Synovial Fluid
*M. hyorhinis* 135S19	Austria	2019	Serosa
*M. hyorhinis* 4236G19	Austria	2019	Joint
*M. hyorhinis* 3741/1/20	Germany	2020	Serosa
*M. hyorhinis* 30S20	Austria	2020	Serosa
*M. hyorhinis* 222S20	Austria	2020	Serosa
*M. hyorhinis* 289S20	Austria	2020	Serosa
*M. hyorhinis* 450S20	Austria	2020	Serosa
*M. hyorhinis* T/0423263	Germany	2021	Lung (BALF ^a^)

^a^ Bronchoalveolar lavage fluid.

**Table 2:** Distribution of the MIC values of 37 *M. hyorhinis* field isolates, including MIC_50/90_ values.

Number of Isolates and MIC Values Obtained (mg/L) *																			
Antimicrobial Agent	0.008	0.015	0.03	0.06	0.12	0.25	0.5	1	2	4	8	16	32	64	128	256	512	MIC_50_	MIC_90_
Gentamicin					-	6	7	16	8	-	-	-	-	-	-	-		1	2
Enrofloxacin	-	-	-	-	-	1	20	14	2	-	-	-						0.5	1
Marbofloxacin	-	-	-	-	-	2	3	25	7	-	-	-						1	2
Florfenicol					1	17	16	1	1	1	-	-	-	-	-	-		0.5	0.5
Clindamycin			-	1	7	10	4	-	-	-	1	1	11	2				0.5	32
Erythromycin		-	-	-	-	-	-	-	1	2	3	9	6	16				32	≥64
Tilmicosin				-	1	2	5	11	3	-	-	-	-	-	-	15		1	≥256
Tulathromycin				9	9	2	1	-	1	-	1	-	2	12				0.25	≥64
Tylosin				5	7	9	-	-	1	-	3	1	4	6	-	1		0.25	64
Tiamulin			7	16	8	5	1	-	-	-	-	-	-	-				0.06	0.25
Doxycycline				13	8	14	1	1	-	-	-	-	-	-	-			0.12	0.25
Tetracycline					23	9	4	-	1	-	-	-	-	-	-	-		0.12	0.5

* Concentrations not included within the test panels are depicted as gray-shaded areas. When no color change was visible, the MIC value was set as equal to or lower than the lowest test concentration. If growth was visible in all tested concentrations, the result was set as equal or higher than the next serially higher MIC value (counts shown as white numbers within gray-shaded areas).

**Table 3:** Distribution of the MIC values of 15 *M. hyorhinis* field isolates obtained during the time period 2002–2012.

Number of Isolates and MIC Values Obtained (mg/L) *																			
Antimicrobial Agent	0.008	0.015	0.03	0.06	0.12	0.25	0.5	1	2	4	8	16	32	64	128	256	512	MIC_50_	MIC_90_
Gentamicin					-	4	2	7	2	-	-	-	-	-	-	-		1	2
Enrofloxacin	-	-	-	-	-	1	6	7	1	-	-	-						1	1
Marbofloxacin	-	-	-	-	-	5	9	1	-	-	-	-						0.5	0.5
Florfenicol					-	5	9	1	-	-	-	-	-	-	-	-		0.5	0.5
Clindamycin			-	-	4	3	1	-	-	-	1	1	4	1				0.5	32
Erythromycin		-	-	-	-	-	-	-	1	-	-	4	3	7				32	≥64
Tilmicosin				-	-	-	1	5	2	-	-	-	-	-	-	7		2	≥256
Tulathromycin				2	5	-	1	-	-	-	-	-	1	6				0.5	≥64
Tylosin				1	3	2	-	-	1	-	2	-	2	2	-	1		8	64
Tiamulin			2	6	4	3	-	-	-	-	-	-	-	-				0.06	0.25
Doxycycline				5	4	5	-	1	-	-	-	-	-	-	-			0.12	0.25
Tetracycline					9	4	1	-	1	-	-	-	-	-	-	-		0.12	0.5

* Concentrations not included within the test panels are depicted as gray-shaded areas. When no color change was visible, the MIC value was set as equal to or lower than the lowest test concentration. If growth was visible in all tested concentrations, the result was set as equal or higher than the next serially higher MIC value (counts shown as white numbers within gray-shaded areas).

**Table 4:** Distribution of the MIC values of 22 *M. hyorhinis* field isolates obtained during the time period 2013–2021.

Number of Tests and MIC Values Obtained (mg/L) *																			
Antimicrobial Agent	0.008	0.015	0.03	0.06	0.12	0.25	0.5	1	2	4	8	16	32	64	128	256	512	MIC_50_	MIC_90_
Gentamicin					-	2	5	9	6	-	-	-	-	-	-	-		1	2
Enrofloxacin	-	-	-	-	-	-	14	7	1	-	-	-						0.5	1
Marbofloxacin	-	-	-	-	-	1	2	17	2	-	-	-						1	1
Florfenicol					1	12	7	-	1	1	-	-	-	-	-	-		0.25	0.5
Clindamycin			-	1	3	7	3	-	-	-	-	-	7	1				0.25	32
Erythromycin		-	-	-	-	-	-	-	-	2	3	5	3	9				32	≥64
Tilmicosin				-	1	3	7	3	-	-	-	-	-	7	1			0.5	64
Tulathromycin				7	4	2	-	-	1	-	1	-	1	6				0.12	≥64
Tylosin				4	4	6	-	-	-	-	1	1	2	4	-			0.25	64
Tiamulin			5	10	4	2	1	-	-	-	-	-	-	-				0.06	0.25
Doxycycline				8	4	9	1	-	-	-	-	-	-	-	-			0.12	0.25
Tetracycline					14	5	3	-	-	-	-	-	-	-	-	-		0.12	0.5

* Concentrations not included within the test panels are depicted as gray-shaded areas. If growth was visible in all tested concentrations, the result was set as equal to or higher than the next serially higher MIC value (counts shown as white numbers within gray-shaded areas).

## Data Availability

All data are contained within the article.
